# Money for medication: a randomized controlled study on the effectiveness of financial incentives to improve medication adherence in patients with psychotic disorders

**DOI:** 10.1186/s12888-014-0343-3

**Published:** 2014-12-02

**Authors:** Ernst L Noordraven, Charlotte H Audier, Anton BP Staring, Andre I Wierdsma, Peter Blanken, Bas EA van der Hoorn, Leona Hakkaart-van Roijen, Cornelis L Mulder

**Affiliations:** Dual Diagnosis Centre (CDP) Palier, Parnassia Psychiatric Institute, The Hague, the Netherlands; Department of Psychiatry, Erasmus MC, Epidemiological and Social Psychiatric Research Institute, Rotterdam, the Netherlands; Bavo-Europoort Mental Health Care, Rotterdam, the Netherlands; Erasmus MC, Institute for Medical Technology Assessment (iMTA), Rotterdam, The Netherlands; Parnassia Addiction Research Centre (PARC), Brijder Addiction Treatment, Parnassia Psychiatric Institute, The Hague, the Netherlands; Altrecht Psychiatric Institute, Utrecht, the Netherlands

## Abstract

**Background:**

Non-adherence with antipsychotic medication is a frequently occurring problem, particularly among patients with psychotic disorders. Prior research has generally shown encouraging results for interventions based on ‘Contingency Management’ (CM), in which desirable behaviour is encouraged by providing rewards contingent upon the behaviour. However, little is known about the application of CM on medication adherence in patients with psychotic disorders. An earlier pilot-study by our study group showed promising results in reducing admission days and increasing adherence. The current study is a randomized controlled trial concerning the effectiveness of a CM procedure called ‘Money for Medication’ (M4M), aimed at improving adherence with antipsychotic depot medication in psychotic disorder patients.

**Methods/Design:**

Outpatients (n =168) with a psychotic disorder will be randomly assigned to either the experimental group (n =84), receiving a financial reward for each accepted antipsychotic medication depot, or the control group (n =84), receiving treatment as usual without financial rewards. Patients are included regardless of their previous adherence. The intervention has a duration of twelve months. During the subsequent six months follow-up, the effects of discontinuing the intervention on depot acceptance will be assessed.

The primary goal of this study is to assess the effectiveness of providing financial incentives for improving adherence with antipsychotic depot medication (during and after the intervention). The primary outcome measure is the percentage of accepted depots in comparison to prescription. Secondary, we will consider alternative measures of medication acceptance, i.e. the longest period of uninterrupted depot acceptance and the time expired before depot is taken. Additionally, the effectiveness of the experimental intervention will be assessed in terms of psychosocial functioning, substance use, medication side-effects, quality of life, motivation, cost-utility and patients’ and clinicians’ attitudes towards M4M.

**Discussion:**

This RCT assesses the effectiveness and side-effects of financial incentives in improving adherence with antipsychotic depot medication in patients with psychotic disorders. This study is designed to assess whether M4M is an effective intervention to improve patients’ acceptance of their antipsychotic depot medication and to examine how this intervention contributes to patients’ functioning and wellbeing.

**Trial Registration:**

NTR2350.

## Background

### Consequences of non-adherence

Approximately 60% of patients with psychotic disorders experience difficulties being adherent over time or fail to take their medications as prescribed, with mean non-adherence rates around 50% [1-3]. Moreover, among patients who do not openly refuse to accept their antipsychotic medication, many are only partially adherent [4]. Failure to take the medication as prescribed is associated with a wide array of adverse individual and societal outcomes such as inconsistent symptom control, more relapses [5-7], more (re)hospitalizations [8,9], more suicide attempts [10,11] and more encounters with police and justice, either as a victim or as a perpetrator [12].

Clinical advantages of antipsychotic medication are often limited by patients’ failure to adhere sufficiently to their prescribed medication. This partial compliance severely reduces the effectiveness of the medical treatment of schizophrenia and interferes with therapeutic efforts [13]. For example, relapses occur within one year for 50 to 75% of the patients with schizophrenia after discontinuing with their antipsychotics [14]. Missing antipsychotic medication has also been associated to double the risk for hospitalization [9]. Throughout this protocol alternative definitions such as ‘acceptance’ or ‘compliance’ relate to the concept of medication adherence.

### Risk factors for non-adherence

Risk factors for non-adherence have been studied extensively and were systematically reviewed by Higashi and colleagues [15]. They distinguished (1) patient-, (2) treatment-, and (3) environmental-related factors to be associated with non-adherence.

1. Patient-related factors included poor insight, negative attitudes towards medication, obesity, previous non-adherence and a shorter duration of illness. Furthermore, comorbid substance use disorders - particularly prevalent in patients with psychotic disorders (70–80%) [16] were also associated with increased non-adherence [9,17-19]. In addition, temperamental characteristics like sensation seeking and disinhibition predicted poor medication adherence in patients with psychotic or mood disorders [20]. From this perspective it is important to also study the (moderating) effects of impulsivity and substance use on the effectiveness of the M4M intervention because certain subgroups of patients could respond differently to the intervention (e.g. impulsive patients perhaps profit less from our intervention since they have more difficulties regulating their behavior). Therefore, this study investigates the role of impulsivity and substance use disorders in patients with psychotic disorders and their associated medication adherence.
2. Treatment-related risk factors for non-adherence included distress by side effects of the medication [21], higher antipsychotic doses and the use of classical antipsychotic medications [22,23].
3. Environmental-related risk factors included stigma of taking medication, lack of support [24], poor therapeutic relationships, financial problems, chaotic living situations and poor aftercare [21,25].

### Interventions to improve compliance

Unfortunately, most studies investigating interventions to improve adherence yield inconsistent results and do not always lead to less symptoms, better functioning or improved quality of life [26,27]. Therefore, a (combination) of innovative methods is needed to help patients take their antipsychotic medication as prescribed [28-30]. One such innovative intervention is contingency management.

Contingency Management (CM) interventions typically reinforce pre-set, well-defined and verifiable target behaviors (e.g., drug abstinence or medication intake), by providing financial incentives or vouchers. Interventions based on CM-principles have been applied in various settings targeting a variety of behaviors, and have shown robust effects in reducing drug use and increasing treatment compliance and medication adherence (for overviews see; [31,32]). Currently, no studies have investigated the effect of CM for non-depot medication adherence in patients with schizophrenia.

In reviewing studies using CM based interventions in patients with mental health problems, Priebe et al. [33] did not find any randomized controlled studies testing the effectiveness of financial incentives to improve depot medication adherence in patients with psychotic disorders. However, two pilot studies were conducted that showed promising results.

Claassen and colleagues [34] included five non-adherent patients of which four patients accepted financial incentives upon medication acceptance. This resulted in improved adherence rates and significantly decreased patients’ hospital admissions during the intervention period. Staring et al. [35], also included five non-adherent patients with psychotic disorders in their pilot study. Results showed that the percentage of accepted depot injections increased from an average of 44% in the previous year to 100% in the year in which financial incentives were offered. While patients had been hospitalized for an average of 100 days in the preceding year, only one patient was re-admitted for 17 days during the intervention year. More recently, the first cluster randomized controlled trial tested the effectiveness of offering financial incentives to patients (n = 141) with psychotic disorders who were partially non-compliant to improve their medication adherence [36]. Interestingly, although adherence to antipsychotic depot medication increased significantly in the CM group as compared to the control group (85% and 69% acceptance of depot after one year), this did not result in a significant difference on clinician rated clinical improvement. In sum, two pilot studies showed promising results and one RCT showed partial positive results of financial incentives upon acceptance of antipsychotic depot medication.

### Study objectives

The goal of the current study is to assess the effectiveness of providing financial incentives upon depot acceptance in psychotic disorder patients. The primary objective of this study is to assess the effectiveness of M4M during the intervention in terms of acceptance of antipsychotic depot medication (the medication possession ratio; MPR). To assess how discontinuing the intervention affects depot acceptance, we will also compare the MPR during the follow-up period (six months), in which no CM takes place. In addition to the MPR, secondary objectives include the longest uninterrupted period of depot medication acceptance, the expired time before depot is taken and attitudes towards medication. Our third objective is to assess the effects of medication acceptance on patients psychosocial functioning, quality of life, cost-utility, substance use and side effects of the antipsychotic medication.

#### Hypotheses

The primary hypothesis is that M4M results in significantly more accepted depots than treatment as usual (TAU). Patients from both the TAU and M4M condition are prescribed antipsychotic depot medication. Secondary hypotheses are that M4M, compared to TAU, leads to (1) longer uninterrupted periods of depot acceptance and (2) less time expired before the depot is taken. From our tertiary measures, we expect M4M (compared to TAU) to result in (3) less severe symptoms and better psychosocial functioning, (4) improved quality of life, (5) less substance use, and (6) lower costs.

Using exploratory analyses we will look for patient characteristics (at baseline) – the stratification variables (gender, comorbid substance use, and medication adherence) and, other variables including impulsivity, motivation and attitudes towards antipsychotic medication and M4M – that could moderate the effects of M4M and might be used for future patient treatment matching. In addition, we will explore the role of potential mediating variables – e.g., medication side effects – in M4M’s effectiveness. Finally, we will analyze self-reported data on patients’ and clinicians’ perceptions of M4M. This enables us to discuss the ethical considerations of M4M.

## Methods

The contents of the study design, data collection, analyses, interpretation of data, writing of the manuscript and the decision to submit the manuscript for publication was not influenced by the funding body (Palier, department of Parnassia Psychiatric Institute).

### Study design

In a parallel-group randomized controlled trial, patients will be randomly assigned to the experimental condition (M4M), or to the treatment as usual (TAU) control condition. Note that during the recruitment phase of the study, only patients who are prescribed or have an indication for antipsychotic depot medication, and who have expressed their willingness to accept antipsychotic depot medication are eligible for inclusion and after providing written informed consent for randomization. Patients assigned to the experimental condition (M4M, n =84) will receive a financial incentive for each prescribed depot they accept, in addition to treatment as usual. Patients in the control condition (TAU, n =84) will receive treatment as usual only without financial incentives upon depot acceptance. After randomization, both patients in the TAU condition and patients in the M4M condition are prescribed depot medication. After the intervention period of 12 months, there will be a follow-up period of 6 months in which patients in both study groups receive TAU and no financial incentives for accepting their prescribed antipsychotic depot medication.

### Participants/Setting

**Figure 1:**
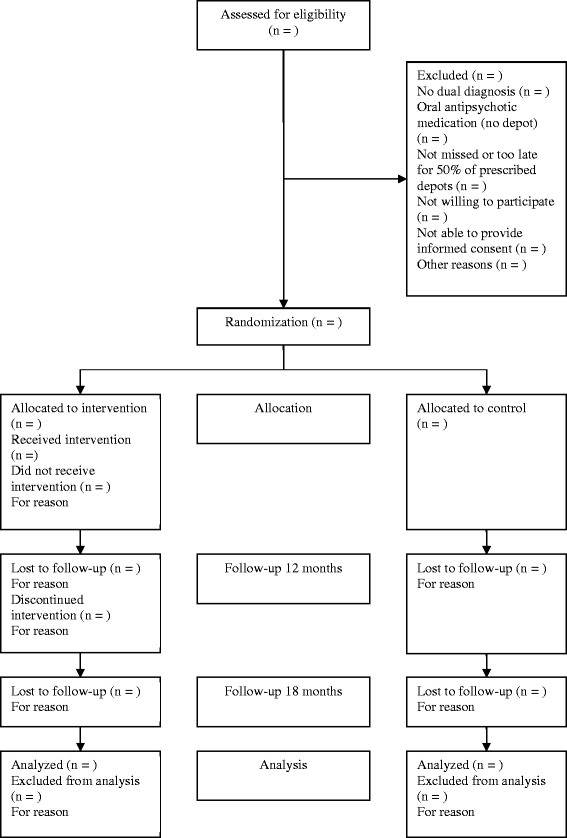
**Participant flowchart.**

Patients will be 168 outpatients with a psychotic disorder from three mental health care institutions in the Netherlands: (1) Palier (‘Dual Diagnosis Centre’ (CDP)), (2) Parnassia and (3) BavoEuropoort. These organizations primarily treat patients with psychotic and other severe mental disorders, (often with comorbid substance use disorder), from the cities of Rotterdam and the Haque in the Netherlands. Per team around two hundred patients with a psychotic disorder are treated. Patients will be recruited based on the following inclusion criteria: age between 18 – 65 years, a psychotic disorder (including schizophrenia, schizoaffective disorder or other psychotic disorders), taking antipsychotic depot medication or an indication to start using depot medication, outpatient treatment (either starting outpatient treatment after discharge from a psychiatric hospital, or being in outpatient treatment for at least four months), and given informed consent. In concordance with their psychiatrist, patients who will start using antipsychotic depot medication are considered to have an indication for antipsychotic depot medication. These patients are - if they meet the other inclusion criteria- eligible to contact for our study. Exclusion criteria are the inability to participate due to cognitive impairments and/or insufficient understanding of the Dutch language (clinical judgment). Refer to Figure 1 Participant flowchart for details.

### Intervention

Patients assigned to the intervention group (M4M; Money for Medication) will receive treatment as usual (see below), plus a financial incentive for each time they accept their prescribed depot of antipsychotic medication during the 12 months experimental study phase. All patients in the M4M group will receive a maximum of 30 euro per month. The amount of money per accepted depot is dependent upon the frequency of depot administration. For example, a patient who receives one depot every two weeks will receive 15 euro per accepted depot. A patient who receives one depot every three weeks will receive 22.50 euro for each accepted depot, et cetera. Patients receiving oral penfluridol with a frequency of once a week will also be included in the study. Oral penfluridol is used when patients have problems taking antipsychotic medication on a daily basis, and when they do not accept intramuscular depot injections. They will receive 7.50 euro for each time they accept their weekly penfluridol oral tablet. The financial incentives will be given by the patients’ treating nurses directly upon administration of the depot or penfluridol. They will receive their depot medication primarily at the clinic (‘depot room’) and sometimes at home during home visits. Patients will sign a proof of receipt.

Patients assigned to the control group will receive treatment as usual (TAU) during the 12 months experimental study phase and during the 6 months follow-up. TAU includes outpatient treatment provided by community mental health teams and flexible assertive community treatment teams [37]. All clinicians encourage continuing depot medication in case this is prescribed by the psychiatrist of the team. When needed, crisis services can be used or patients can be hospitalized (in)voluntarily. The type and dosage of the depot antipsychotic medication and other medications patients receive will be determined by the patients’ psychiatrist together with the patient. The type, frequency and dosage will not be affected by participation in the study. Administration of the depots will be done by the psychiatric nurses working in the teams.

### Procedure

Candidate participants will be selected from the caseloads, applying the in- and exclusion criteria. Patients who meet the criteria will be informed and asked to participate by their clinician. Patients who consider participation receive a take-home brochure with information about the study. The clinician asks the patient’s permission to be contacted by a researcher. If the patient agrees, the researcher contacts the patient to schedule an appointment for the baseline interview. If a patient indicates that he or she does not want to participate, this will be registered anonymously together with their demographic and clinical characteristics (DSM IV-TR diagnosis on axes I and II) to enable assessment of selection bias. If possible, the patient will be asked to explain why he or she does not want to participate.

With support of the management, all teams and their clinicians have expressed their willingness to co-operate with the conduct of our Money for Medication study. Clinicians of course can be resilient about the concept and intervention of our study. Therefore, we assess clinicans’ attitudes towards M4M.

Prior to the baseline interview the researcher explains the design and purpose of the study, the research goals and the randomization procedure. After written informed consent is given, the baseline interview will take place and subsequently, participants will be randomized to the intervention (M4M) or control condition (TAU). Randomization will be stratified by site, gender, substance use disorder (absent vs. prevalent) and previous compliance with antipsychotic medication (compliance rate <50% vs. ≥50%). There will be three interviews at 0, 12 and 18 months (see Table 1). All participants will receive a remuneration of 20 euro for each interview. In the cases where the researchers cannot overcome certain practical obstacles (e.g. imprisonment, hospitalization), patients who can demonstrate that they have accepted their depot medication (for instance in the form a written statement by the treating prison or hospital medical doctor), receive their monetary reward as soon as possible, but with a delay. In case of discontinuation of depot intake, the monetary reward will stop and data of non-depot medication intake will be monitored in order to have a complete overview on the number of patients discontinuing depot medication and switching to non-depot.

**Table 1:** **Measures and instruments and assessment times**

**Category**	**Outcome measure**	**Instrument**	**Assessment (month)**		
			**0**	**12**	**18**
**Demographics**		Registration forms	X		
**Medication acceptance**	- Percentage accepted depots (MPR)	Registration forms	Continuously	Continuously	Continuously
	- Longest uninterrupted period	Registration forms	Continuously	Continuously	Continuously
	- Time expired before depot is taken	Registration forms	Continuously	Continuously	Continuously
	- Attitudes towards medication	ROMI	X	X	X
**Psychosocial functioning**	- Psychiatric symptomatology	PANSS	X	X	X
	- Health, psychological and social functioning	HoNOS	X	X	X
	- Substance use	ASI	X	X	X
		CIDI-SAM	X		
		Urine screens	X	X	X
	- Quality of Life	MANSA	X	X	X
	- Antipsychotic side-effects	ASC	X	X	X
	- Treatment Entry Questionnaire	TEQ	X	X	X
	- Dickman Impulsivity questionnaire	DII	X	X	X
**Cost-utility**	- Health-care consumption	TiC-P	X	X	X
	- Health-related quality of life	EQ-5D	X	X	X
	- Self-reported delinquent behaviour	SRD	X	X	X
	- Effort of clinicians	Registration forms	Continuously	Continuously	Continuously
**Ethical aspects**	- Attitudes towards M4M	Questionnaire constructed for the current study	X	X	X

ROMI: Rating Of Medication Influences, PANSS: Positive And Negative Symptoms Scale, HoNOS: Health of the Nations Outcome Scales, ASI: Addiction Severity Index, CIDI-SAM: Composite International Diagnostic Interview – Substance Abuse Module, MANSA: Manchester Short Assessment of Quality of Life, ASC: Antipsychotic Side-effects Checklist, TEQ: Treatment Entry Questionnaire, TiC-P: Trimbos/iMTA questionnaire for Costs associated with Psychiatric Illness, EQ-5D: EuroQol-5D, Quality of Life, SRD: Self-Reported Delinquency questionnaire.

Originally the start date for patient recruitment was May 21, 2010 and was planned to be completed by September 2012. Due to a change in personnel and organizational factors that caused logistical delays, patient recruitment was low and continued again in September 2013. Note that this is an ongoing study and that we expect to finish our complete data collection by April 2016. Therefore, we expect to submit the results of this study in 2016.

### Instruments

#### Baseline variables

Demographic variables, DSM-IV diagnoses on Axis I and II, and psychiatric history (including hospitalizations during the last three years, current antipsychotic and concomitant medication, and antipsychotic depot acceptance 4 months before the study) will be collected in the first interview and from patients’ records.

### Outcome measures

- *Primary outcome measure:* The primary outcome measure is medication acceptance, represented by the percentage of accepted depot injections. This is defined as the ‘Medication Possession Ratio’ (MPR) first reported by Sclar, Chin and Skaer [38]. The MPR is the number of accepted depots antipsychotic medication divided by the number of prescribed depots antipsychotic medication (the number of supplies needed for continuous use of antipsychotic medication).
- *Secondary outcome measures:* The secondary outcome measures include additional measures of adherence, including the longest uninterrupted period of depot medication acceptance, the time expired before the depot is taken and patients attitudes towards medication.
- *Longest uninterrupted period of depot medication acceptance:* Sometimes occasional missed doses are not regarded as ‘non-adherence’ [39]. However, as even partial adherence can severely undermine clinical improvement [9] it is important to strive for continuous medication adherence. Therefore, the longest uninterrupted period of medication acceptance will be assessed as well. In sum, this outcome measures the time period (number of days/weeks) a patient takes the prescribed antipsychotic depot medication according to schedule, without missing or not taking a single depot prescription).
- *Time expired before depot is taken:* Following Priebe et al. [33], we will monitor the time that has expired before the patient accepts the prescribed depot. Note that all patients receive depot medication (M4M and TAU) according to their own schedule (i.e., every 14 days). This variable (*time expired before depot is taken*) allows us to see whether patients are late for their prescribed depot. The time ‘slippage’ of taking depots is defined as the percentage of the prescribed time interval that has expired before the depot is taken.
- *Attitudes towards medication:* To assess how patients attitudes towards medication relate to the effectiveness of M4M, the ‘Rating of Medication Influences’ (ROMI) scale [40] will be used. The ROMI measures attitudinal and behavioral factors influencing patient adherence with neuroleptic treatment. The ROMI consists of three subscales related to adherence (prevention, influence of others and medication affinity) and five subscales related to non-adherence (denial/dysphoria, logistical problems, rejection of label, family influence and negative therapeutic alliance). In sum, the ROMI asks questions about the reasons for taking medication and patients’ general attitudes towards treatment. An example item: “Do you have a positive relation with the clinical staff?” Patients can answer yes/no and indicate to what extent this affects their medication intake (no/some/strong).
- *Tertiary outcome measures:* The tertiary outcome measures include measures on the effects of medication acceptance on patients psychosocial functioning, substance use, quality of life and side-effects of the antipsychotic medication:
- *Psychiatric symptomatology:* Psychiatric symptomatology will be assessed by trained interviewers with the Dutch version of the ‘PANSS’ , the Positive and Negative Syndrome Scale, originally conceived by Kay, Fiszbein and Opler [41]. The PANNS consists of three subscales: positive symptoms (7 items), negative symptoms (7 items) and general psychopathology (16 items, including anxiety and depression). Items are scored on a scale from 1 (symptom absent) to 7 (symptom interferes with almost all aspects of daily functioning). Internal and external consistency of the PANNS has been found to be adequate [42-44].
- *Health, psychological and social functioning:* To assess patients’ health and psychosocial functioning, the Dutch translation of the Health of the Nations Outcome Scales (‘HoNOS’) [45,46] will be administered by trained interviewers. The HoNOS is a structured interview to quantify health and social functioning during the last two weeks on four subscales (behavioural problems, impairments, symptoms and social problems). Items are rated on a 5-point scale ranging from 0 (no problems) to 4 (severe to very severe problems).
- To test for the potential modifying effect of impulsivity on the M4M intervention, we will assess impulsivity by means of the Dickman Impulsivity Inventory (DII) [47], which has been validated for the Dutch situation [48] and has good psychometric properties among substance users as well [49]. The DII consists of 24 dichotomous items, resulting in a “functional impulsivity” and a “dysfunctional impulsivity” score.
- *Substance use:* Substance use will be assessed with the ‘Alcohol and drug use’ section of the European version of the ‘Addiction Severity Index’ (‘EuropASI’) [50] and the Substance Abuse Module of the International Diagnostic Interview [51]. The CIDI is a structured interview based on the criteria and definitions of the ICD-10 and DSM-IV with good psychometric properties [52]. Self-reported drug-use will be verified by urinanalysis sticks at baseline, 12 and 18 months (follow-up) for amphetamines, benzodiazepines, cocaine, morphine/heroin and cannabis.
- *Subjective Quality of Life:* To assess patients’ subjective quality of life we will use the third section of the ‘Manchester Short Assessment of Quality of Life’ (‘MANSA’) [53]. The MANSA assesses the patients subjective ratings of life in general and satisfaction with several more specific domains of quality of life, including work or education related issues, financial situation, social relations, leisure activities, accommodation, family situation, personal safety and physical and mental health. Items are rated on a seven-point scale ranging from 1 (could not be worse) to 7 (could not be better). The MANSA has good psychometric properties [53,54].
- *Antipsychotic Side-effects:* To assess how medication affects patients’ subjective wellbeing, we will use the 17-item Dutch translation of the ‘Antipsychotic Side-effect Checklist’ (‘ASC’) [55]. The ASC-C is a checklist designed for mental health clinicians to use as a brief interview to check for common problems (side-effects) associated with the use of antipsychotic medication during a regular therapeutic session. Items are rated as: symptom present or symptom absent.
- *Cost-utility:* The cost-utility of M4M will be compared with treatment as usual. To estimate direct health care from a societal perspective, costs will be determined and calculated by multiplying resource use with official charge standards. Our focus will be on patients’ health care consumption (admissions, contacts with clinicians, and efforts initiated to provide depots) and illegal activities. Measures will be collected from the patients file and the Trimbos/iMTA self-report questionnaire for Costs associated with Psychiatric Illness (TiC-P; [56]), a questionnaire for Self-Reported Delinquency (SRD, adapted from the INternational CAnnabis Need of Treatment (INCANT) study), and the depot acceptance registration forms.
- *QALY’s will be assessed using the EQ-5D:* The EQ-5D is a standardized instrument that scores health-related quality of life on five levels of health (mobility, self-care, daily activities, pain/discomfort and anxiety/depression), which generates a score for health-related quality of life that can be used as a weight to calculate Quality Adjusted Life Years or ‘QALY’s’ [57], a weighted health-index. The EQ-5D has been shown to have good discriminative and construct validity and to be sensitive in detecting changes in QoL ratings in patients with substance use [58].
- *Time spent by clinicians to provide depot:* To assess how M4M affects patients’ willingness to accept their antipsychotic depot medications, the time and effort spent by the clinicians to provide the depot (e.g. calling, home visits, et cetera) will be monitored with standard registration forms designed for the current study.
- *Attitudes towards M4M:* In addition to the outcome measures above, patients’ and clinicians’ attitudes towards M4M will be assessed with a short questionnaire constructed for the current study. Items address different attitudes towards M4M in terms of its advantages and disadvantages (e.g. effects on motivation, insight, wellbeing, depot acceptance, dependency, the relationship between the patient and the clinician, and moral, ethical and practical considerations). Items will be scored on a 5-point scale (1 = strongly disagree, 5 = strongly agree).
- *Intrinsic and extrinsic motivation:* To measure patients” intrinsic and extrinsic motivation during the study, the Dutch version of the Treatment Entry Questionnaire (TEQ) is being used, which has good psychometric properties [59]. On 27 statements regarding motivation for the current intervention patients answer if they agree (1 = strongly disagree, 7 = strongly agree).

### Ethical approval

The study protocol has been approved by the accredited Dutch Medical Ethical Trial Committee (METC) of the Erasmus University Medical Centre (registered under number NL31406.097.10 and file number P13.258). According to the Dutch Data Protection Act (DPA) data will be safely stored and anonymized and is only accessible for members of the research group or the Medical Ethical Committee. All patients will provide informed consent before entering the study.

### Sample size/power

Following the CONSORT statement we calculated our power to the primary outcome measure of this study and not for the secondary or tertiary outcome measures. Based on previous findings and study protocols [33,60], we expect a difference of 65 to 85 percent (an absolute difference of 20%) of accepted depots between the control group and the money-for-medication condition. In terms of Cohen’s h, this constitutes a medium effect size (h =0.5). With Type I error rate (alpha) set at 5%, power at 0.90 (1 – Type II Error rate; 1-β), and 20—25% drop-out, we will need 84 patients per arm to detect an absolute difference of 20% [61]. In total 168 patients will be included.

### Statistical analyses

The primary outcome will be reported as accepted depots as percentage of planned depots, most often weekly, biweekly or monthly. The effects of the intervention on our outcome measures will be analysed using generalized linear models as appropriate to the outcome, with random effects for sites or treatment teams. Sensitivity analyses will be conducted to explore the impact of different strategies for handling missing data. A detailed analysis plan will be completed prior to analysis of baseline measurements.

## Discussion

The aim of the current randomized controlled study is to assess the effectiveness of financial incentives (M4M), compared to treatment as usual, in improving the acceptance of antipsychotic depot medication in patients with psychotic disorders. Our primary outcome measure will be the MPR. Secondary outcome measures include patients’ health, social and psychological functioning and subjective quality of life. Tertiary measures are used to assess the effects of medication acceptance on patients’ psychosocial functioning, substance use, quality of life and side effects of the antipsychotic medication. This allows us to assess not only whether M4M is an effective intervention to improve acceptance, but also to what extent medication acceptance contributes to patients’ wellbeing We will also compare the MPR during the follow-up period (six months), in which no CM takes place.

### The use of financial incentives in M4M

In the pilot study of Claassen et al. [34], M4M did not have a negative impact on the therapeutic relationship. Furthermore, they have not found that other patients who did not participate in the M4M study complained about unequal treatment or demanded to be paid for taking medication as well.

In the pilot study of Staring, Mulder, and Priebe [35], all five participants considered M4M to be a good project. The reasons they gave to participate in the pilot were “I don’t like the injection, but money makes it better”, “Money keeps me motivated”, and “The depot injections keep me balanced”. When prompted, two patients said that they perceived financial incentives as a voluntary and non-coercive measure, two patients did not know what to think about this, and one indicated that he perceived financial incentives as a coercive measure, saying that “I have to take the medication anyway”. All patients said that they spent the money on food and cigarettes, and one patient also bought household products. It was observed, however, that at least one patient had spent some of the money on cannabis. Other patients did not ask to be offered incentives as well and no negative impacts on therapeutic relationships were noted. Some patients however felt that they should receive more money (they received 10 euro for every two weekly depot, 15 euro for every three-weekly depot and 20 euro for every four-weekly depot).

Although higher payments have been found to result in bigger effects [62,63], in the present study we have chosen a maximum of 30 euro’s on average per month because (1) patients could get used to or become financially dependent on higher payments or even lose their social security benefits and (2) to strive for acceptable cost-utility of M4M. From a societal perspective it is important to focus on cost-effective interventions and given the results of prior research [35], it seems not necessary to use higher incentives.

### Strengths and limitations

Our study started in 2010, prior to some of the recent findings as described above. The difference with the earlier RCT studying the effects of M4M [36] is that we will include both patients who are partially non-compliant, as well as patients who are compliant in taking depot medication. The rationale to also include compliant patients is the observation in several studies that around thirty percent of patients initiated on antipsychotic depot medication cease to accept their depot within one year [5,64,65]. In addition, when we eventually might want to implement this intervention into daily clinical practice, it is more ethical as well as more practical to reward both compliant and non-compliant patients.

A possible limitation of this study are the different medication depots. Although most patients receive medication injections it is also allowed for patients to take oral penfluridol. The disadvantage of this oral antipsychotic medication is that it is more difficult to check if patients actually take their medication. However, penfluridol is also a depot and because we aim to test if our intervention is broadly applicable we decided to include both patients with oral penfluridol and injections.

Another limitation is that the clinicians cannot be blinded to the intervention condition, possibly resulting in a more stimulating attitude for accepting depot medication in the intervention group. Also, the interviewers are not blind to the patients’ condition and therefore can rate patients’ responses as more positive or negative.

Furthermore, the intervention effect can be influenced by different depot frequencies. For example, patients who receive money every week are rewarded four times as often compared to patients that receive depot every month. Receiving a small incentive more frequently can be more stimulating or motivating compared to receiving a bigger incentive only once a month. This can interfere with our intervention effect, even though the mean amount of money per month remains equal for all participants.

### Ethical issues

Ethical concerns have been raised about paying patients to accept their medication and whether this is an acceptable means in the treatment of patients with psychotic disorders [66,67]. One of these concerns is that patients’ intrinsic motivation to accept medication will disappear if money is involved. We will study this by assessing intrinsic motivation over time, as possible decreases in depot acceptance can occur during the 6 month follow-up without M4M. Another frequently raised ethical argument is that patients might buy drugs or alcohol from the money they receive. We will monitor alcohol and drug use by using assessment scales as well as obtaining urine samples.

Apart from these ethical concerns, we will also assess the intervention from a cost-utility perspective, because this is an important factor to consider from a societal point of view. In conclusion, we will test if M4M improves patients’ MPR, reduces their psychotic symptoms and contributes to a clinical improvement.
